# The involvement of aldosterone on vascular insulin resistance: implications in obesity and type 2 diabetes

**DOI:** 10.1186/1758-5996-6-90

**Published:** 2014-08-24

**Authors:** Thiago Bruder-Nascimento, Marcondes AB da Silva, Rita C Tostes

**Affiliations:** Department of Pharmacology, Ribeirao Preto Medical School, University of Sao Paulo, Av Bandeirantes 3900, Ribeirao Preto, SP 14049-900 Brazil

**Keywords:** Aldosterone, Insulin resistance, Vascular dysfunction, Cardiovascular disease

## Abstract

Aldosterone, a mineralocorticoid hormone produced at the adrenal glands, controls corporal hydroelectrolytic balance and, consequently, has a key role in blood pressure adjustments. Aldosterone also has direct effects in many organs, including the vasculature, leading to many cellular events that influence proliferation, migration, inflammation, redox balance and apoptosis.

Aldosterone effects depend on its binding to mineralocorticoid receptors (MR). Aldosterone binding to MR triggers two pathways, the genomic pathway and the non-genomic pathway. In the vasculature e.g., activation of the non-genomic pathway by aldosterone induces rapid effects that involve activation of kinases, phosphatases, transcriptional factors and NAD(P)H oxidases.

Aldosterone also plays a crucial role on systemic and vascular insulin resistance, i.e. the inability of a tissue to respond to insulin. Insulin has a critical role on cell function and vascular insulin resistance is considered an early contributor to vascular damage. Accordingly, aldosterone impairs insulin receptor (IR) signaling by altering the phosphatidylinositol 3-kinase (PI3K)/nitric oxide (NO) pathway and by inducing oxidative stress and crosstalk between the IR and the insulin-like growth factor-1 receptor (IGF-1R).

This mini-review focuses on the relationship between aldosterone and vascular insulin resistance. Evidence indicating MR antagonists as therapeutic tools to minimize vascular injury associated with obesity and diabetes type 2 is also discussed.

## Aldosterone

For a long time, aldosterone was simply considered a hormone that regulates renal function and corporal hydroelectrolytic balance/plasma osmolality and, consequently, extracellular volume and blood pressure [1, 2]. However, aldosterone influences the function of many other organs including the brain, the heart, and the vasculature [1, 3].

Aldosterone is mainly synthesized by the adrenal glands, at the glomerular zone. Aldosterone binds to mineralocorticoid receptors (MR), a cytoplasmic receptor that operates as a transcription factor regulating gene and protein expression, the so-called genomic pathway. Furthermore, aldosterone rapidly activates many other signaling pathways, or non-genomic pathways, which are not sensitive to translation or transcription inhibitors [4, 5]. These quick events are described as being associated to the classic MR or to a membrane aldosterone receptor, the G protein-coupled receptor 30 (GPR30) [6]. MR are expressed not only in the kidneys, but also in extrarenal tissues, such as adipocytes, cardiomyocytes, macrophages, endothelial cells (EC) and vascular smooth muscle cells (VSMC) [7–9]. In these cells, aldosterone activates inflammatory, proliferative and migratory processes, as discussed below [7, 10, 11].

In the vasculature, aldosterone activates several signaling proteins including mitogen-activated protein kinases (MAPK) such as extracellular signal-regulated kinases 1 and 2 (ERK1/2), p38 and c-Jun N-terminal kinase (JNK) [12–14], Rho kinase [11], transcriptional factors like the nuclear factor kappa-light-chain-enhancer of activated B cells (NF-kB) [15], adhesion molecules including vascular cell adhesion molecule 1 (VCAM-1) and intercellular adhesion molecule 1 (ICAM-1), and the non-receptor tyrosine kinase protein c-Src [7], which then trigger other signaling pathways. c-Src, for example, activates the nicotinamide adenine dinucleotide phosphate (NADPH) oxidases (Noxes) through p47phox phosphorylation, leading to the generation of reactive oxygen species (ROS) [16] and to further activation of redox-sensitive proteins [7, 17, 18].

Due to its many effects in the cardiovascular system, aldosterone plays a critical role in cardiovascular diseases (CVD) as well as in metabolic diseases including insulin resistance, diabetes type 2 (DM2) and obesity [1, 19]. Accordingly, epidemiological studies demonstrate a positive relationship between increased aldosterone levels and enhanced rates of CVD [20, 21] and metabolic diseases [1, 19, 22–30].

Angiotensin II (Ang II), the most powerful biologically active product of the Renin-Angiotensin-Aldosterone system (RAAS), stimulates aldosterone secretion and cell growth in adrenocortical cells [31]. The RAAS plays a major role in the genesis and progression of CVD, including arterial hypertension, myocardial infarction and stroke. Of importance, aldosterone contributes along with Ang II to the adverse actions of the RAAS in CVD [32]. Accordingly, treatment of patients with cardiovascular risk like diabetic patients, with antagonists of the Ang II type 1 receptor (AT1) or with inhibitors of the Angiotensin Converting Enzyme (ACE) importantly reduce cardiovascular risks [33, 34]. In addition, MR antagonists, such as eplerenone and spironolactone, also have beneficial effects in patients with CVD, as discussed below [35, 36].

Clinical trials, as the Randomized Aldactone Evaluation Study (RALES), have shown that daily treatment with 25 mg of spironolactone substantially reduces the risk of both morbidity and death among patients with severe heart failure. In addition, after eight weeks of treatment, if the patient showed signs or symptoms of progression of heart failure, the dose of spironolactone could be increased to 50 mg without evidence of hyperkalemia [36].

In the Eplerenone Post-Acute Myocardial Infarction Heart Failure Efficacy and Survival Study (EPHESUS) clinical trial, patients were randomly assigned to eplerenone (25 mg per day initially, until to a maximum of 50 mg per day) or placebo. The addition of eplerenone to optimal medical therapy reduced morbidity and mortality among patients with acute myocardial infarction complicated by left ventricular dysfunction and heart failure [37]. In hypertensive patients, eplerenone treatment, compared with an atenolol regimen, reduced proinflammatory mediators, such as macrophage chemoattractant protein 1 (MCP-1), osteopontin, basic fibroblast growth factor (bFGF), and inteleukin-8 (IL-8), as well as stiffness of subcutaneous small resistance arteries [38]. An important finding in clinical studies with MR antagonists is that reduction of cardiovascular risks does not depend on blood pressure changes [39, 40].

Aldosterone has also been implicated in the development of insulin resistance. One interesting study published by Catena et al. [41] showed that patients with tumoral and idiopathic aldosteronism present insulin resistance, and that both surgical treatment and treatment with aldosterone antagonists rapidly and persistently restore sensitivity to insulin. A positive association between increased plasma aldosterone concentrations with plasma glucose, insulin, C-peptides, and HOMA (Homeostasis Model Assessment, which estimates steady state β cell function and insulin sensitivity) has also been reported in a population of patients with essential hypertension [42].

A positive correlation between fasting insulin and plasma and urinary aldosterone levels was demonstrated in patients with class II–IV heart failure included in the ALOFT (Aliskiren Observation of Heart Failure Treatment) study. In addition, early-morning fasting insulin, homeostasis model assessment of insulin resistance (HOMA-IR), and insulin/glucose ratio (IGR) were higher in patients with aldosterone escape and high urinary aldosterone excretion, when compared to the healthy population [43]. Furthermore, in experimental models of obesity (ob/ob and db/db mice), eplerenone treatment also reduced the high levels of glucose, HOMA-IR, and plasma triglyceride concentration, and increased adiponectin levels [44].

In cultured adipocytes, basal and insulin-stimulated glucose uptake is decreased by high aldosterone concentrations, an effect prevented by RU486, an antagonist of glucocorticoid receptors. Surprisingly, eplerenone did not abolish these effects, indicating that aldosterone has MR-independent effects [45]. In addition, a strong relationship between genetic variants of the CYP11B2 gene, which encodes for aldosterone synthase, and glucose plasma levels has been reported [46].

## The relationship between obesity, insulin resistance and aldosterone secretion

Recently, Briones and colleagues showed that aldosterone is also produced by the perivascular adipose tissue (PVAT) [9]. Their study showed that adipocytes express aldosterone synthase and produce aldosterone in an Ang II/AT1/calcineurin/nuclear factor of activated T-cells (NFAT)-dependent manner. Interestingly, adipocyte-derived aldosterone regulates adipocyte differentiation and vascular function in an autocrine and paracrine manner, respectively. These findings indicate that adipocytes may represent an important source of aldosterone as well as the putative link between aldosterone and vascular dysfunction in metabolic diseases, such as diabetes mellitus and obesity. Goodfriend and colleagues have previously suggested that aldosterone can be released by visceral fat in obese male and female voluntaries [47]. These authors also found that certain fatty acids stimulate aldosterone production *in vitro* by rat adrenal cells incubated with rat hepatocytes, but not in adrenal cells alone, suggesting that fatty acids from visceral adipocytes induce hepatic formation of an adrenal secretagogue [48].

Leptin plays a crucial role on body fat gain. Leptin increases energy expenditure and induces satiety [49]. Obese and insulin-resistant patients exhibit higher leptin plasma levels than control subjects, i.e. they become leptin-resistant, exhibiting a loss of leptin effects. There is a controversy regarding the effects of leptin on aldosterone release. For example, renin and aldosterone levels do not change after treatment of rats with leptin [50]. In addition, leptin infusion leads to natriuresis and diuresis [51] and stimulation of primary adrenal cell cultures with leptin inhibits adrenocortical steroid production [52].

On the other hand, increased plasma renin activity is observed in rats chronically treated with leptin [53]. Furthermore, Belin de Chantemelle and colleagues showed that plasma aldosterone levels are increased in obese mice and further increase with sustained leptin infusion, and the chronic α1-adrenergic receptor antagonism with prazosin blunted obesity-induced increased aldosterone levels and also abolished leptin-stimulated aldosterone secretion in obese mice [53]. These data indicate that obesity-associated increased renin activity and leptin-stimulated aldosterone production may result from increased sympathetic activity. These results also suggest that leptin may influence aldosterone secretion and perhaps participate in obesity- and type 2 diabetes-associated insulin resistance. Accordingly, aldosterone would be an interesting target to minimize insulin resistance- and/or obesity-associated deleterious effects.

Reinforcing this suggestion, aldosterone has been shown to inhibit insulin effects in the vasculature, i.e. aldosterone induces vascular insulin resistance [19]. Although still unclear, some potential mechanisms for aldosterone-induced insulin-resistance are already described, including desensitization of proteins involved in insulin effects, such as Insulin Receptor Substrate (IRS)-1, Phosphatidylinositide 3-Kinase (PI3K), Akt and nitric oxide synthase (NOS). Oxidative stress, possibly mediated by increased NAD(P)H-oxidase activity, as well as hybridization of insulin receptor (IR) and insulin-like growth factor-1 receptor (IGF-1R) are also candidate mechanisms [1, 2, 19].

The relationship between aldosterone and vascular insulin resistance is discussed in detail in the next section. Evidence suggesting the MR antagonists as therapeutic tool to minimize vascular injury associated with obesity and diabetes type 2 is also discussed.

## Insulin resistance

Pancreatic β-cell dysfunction plays an important role in the pathogenesis of DM1 and DM2. Insulin is a peptide hormone composed of 51 amino acids that is synthesized, packaged, and secreted in pancreatic β cells. It was the first protein whose primary structure was elucidated. This feat was accomplished by Fred Sanger and led to a Nobel Prize [54, 55].

Insulin is synthetized in the pancreatic β cells as preproinsulin and then processed to proinsulin, whose structure is stabilized by three disulfide bonds. In the Golgi apparatus insulin is sorted into secretory vesicles, where it is converted to insulin and C-peptide. These peptides are stored in organized mature secretory vesicles/granules, awaiting their regulated on-demand discharge into the bloodstream [56–58]. Insulin is secreted primarily in response to glucose, while other nutrients such as free fatty acids and amino acids can augment glucose-induced insulin secretion. In addition, various hormones, such as melatonin, estrogen, leptin, growth hormone, and glucagon like peptide-1 also regulate insulin secretion. In these cases aerobic glycolysis and mitochondrial oxidation produce metabolic signals, including a rise in the ATP to ADP concentration ratio. This closes ATP-dependent potassium (K^+^-ATP) channels, leading to depolarization of the plasma membrane, which causes calcium (Ca^2+^) influx that stimulates insulin exocytosis [56–58]. β cells are especially adapted to support these processes in the face of varying demands. However, high-level stimulation of insulin synthesis, such as in diabetes, may lead to β cells damage or death [56–58].

Insulin receptors are composed of two extracellular α-subunits that are each linked to a β-subunit and to each other by disulfide bonds. Reduction of the bonds that link the α-subunits produces a α-β monomer that binds insulin with reduced affinity and is devoid of insulin-stimulated tyrosine kinase activity [58–60]. Reconstitution of such hetero-dimers into hetero-tetramers restores both high affinity insulin binding and insulin-stimulated kinase activity [58–60].

The main effects of insulin in the organism include glucose uptake in muscle and adipose tissues, glycolysis, glycogen synthesis, protein synthesis and uptake of ions, including K^+^. Otherwise, insulin blocks glucogenesis, glycogenolysis, lipolysis and proteolysis [56–58]. Insulin also contributes to nutrient and hormone delivery to skeletal muscle by increasing blood flow and recruiting capillaries [61]. Insulin-induced vasodilatation and capillaries recruitment in skeletal muscle seem to depend on NO, since a nitric oxide inhibitor, L-NG-Nitroarginine Methyl Ester (L-NAME), inhibits the microvascular effects of insulin [62]. In addition to promote endothelium-dependent vasodilation and NO release, insulin also stimulates production of vasoconstrictors agents, such as endothelin-1 (ET-1), mediated by Ras/MAPKs signaling [63, 64]. Although there are controversies on insulin effects, it is suggested that insulin-induced NO production limits the contractile, proliferative, and inflammatory actions of insulin-stimulated growth factor production [65, 66].

Aldosterone may impair insulin-signaling pathway in skeletal muscle by different mechanisms: by reducing NO bioavailability and Akt activity, by increasing the sources and production of ROS and insulin-like growth factor-1 (IGF-1) signaling pathway. These mechanisms are detailed in the next section, but with emphasis on the role of aldosterone on vascular insulin resistance.

In the vasculature, insulin directly promotes vasodilation through changes in Ca^+2^ sensitivity and Ca^+2^ handling mechanisms in VSMC, and indirectly through activation of PI3K/inducible NO synthase (iNOS)/cyclic guanosine monophosphate signaling, and phosphorylation of Akt or PKB in the endothelial cells [67, 68]. Thus, insulin induces vasodilation mediated by IRS/PI3K signaling both in VSMC and EC.

Resistance to insulin signaling is a cardiovascular risk factor that underlies the pathophysiology of the metabolic syndrome. Although insulin in compensatory hyperinsulinemia has adverse mitogenic and proinflammmatory effects, at normal physiologic concentrations, preserved sensitivity to insulin signaling has a protective effect in the vasculature [69].

Insulin resistance is characterized by the inability of a tissue to respond to insulin, which will impair the input of glucose into the cell as well as the sensitivity of IRS/PI3K/NO signaling pathway [64, 68]. Vascular insulin resistance is considered an early contributor to vascular damage [40]. Under insulin resistance conditions, the antioxidant, anti-inflammatory, anti-atherogenic properties of insulin, as well as its ability to induce PI3K/NO-dependent vasodilation are attenuated, prevailing its deleterious effects [30, 64, 68, 70, 71].

## The role of aldosterone on vascular insulin resistance

There is growing interest in the role of aldosterone and its receptors in the pathogenesis of insulin resistance [71]. As already discussed, evidence points that aldosterone plays an important role on the metabolic syndrome [1, 19]. Elevated levels of aldosterone are present in obese and insulin-resistant patients and rodent models [23–25], leading to proliferation, inflammation, oxidative stress, contributing to impaired insulin signaling, decreasing glucose transport, inducing vascular dysfunction and cardiovascular abnormalities [26–30, 70, 71].

In 3T3-L1 adipocytes, aldosterone blocks insulin-induced glucose uptake and induces degradation of IRS-1 and 2, an effect that depends on ROS generation; in human adipocytes aldosterone also impairs insulin sensitivity, implying that aldosterone induces insulin resistance in the adipose and vascular tissues [70, 71].

Aldosterone exerts negative effects on structural and functional integrity of the pancreatic β-cell by favoring inflammatory and oxidative stress conditions, which lead to decreased insulin release and actions, including actions in the vasculature [72].

A model of dietary salt restriction, associated with increased aldosterone production, exhibits impaired insulin-induced vasodilation, as well as increased systemic insulin resistance [73], indicating that even mild elevations in plasma aldosterone produce significant effects on vascular insulin sensitivity and may influence cardiovascular outcomes [40]. Furthermore, rats infused with aldosterone also develop systemic and vascular insulin resistance, effects that might be related to increased levels of insulin-like growth factor-1 receptor (IGF-1R) and to hybridization of IGF-1R and IR [71]. Since these effects are prevented by a MR antagonist and tempol, an antioxidant agent, a role for MR receptor and ROS generation, probably from NAD(P)H oxidase source, has been suggested [70, 71]. Compared with IR, IGF-1R is more abundant in VSMCs, and expression of IGF-1R is increased in aortas of diabetic animals. Furthermore, subunits of IR and IGF-1R easily build hybrid receptors that have higher affinity for IGF-1 than for insulin. IGF-1 induces hypertrophic changes and insulin resistance via IGF-1R in the vasculature. Although the affinity of IGF-1R for insulin is lower compared with IGF-1, high concentrations of insulin, which are often observed in patients and animal models with insulin resistance, may affect intracellular signaling pathways dependent on IGF-1R or hybrid receptors [74–78].

Signaling mechanisms by which insulin regulates endothelial NO production have been substantially clarified. Insulin receptor phosphorylation of IRS-1, which binds and activates PI3K, leads to activation of 3-phosphoinositide-dependent protein kinase-1 (PDK-1), which in turn phosphorylates and activates Akt. Akt directly phosphorylates eNOS at Ser^1177^, resulting in increased eNOS activity and NO production. Endothelium-derived NO diffuses into adjacent vascular smooth muscle, where it evokes vasorelaxation [65, 79–81].

Aldosterone can affect insulin-induced eNOS activation at different points. Aldosterone increases IGF-1R, which is an important negative regulator of insulin sensitivity in the endothelium. This has been confirmed by deletion of IGF-1R, which culminates in increase in NO bioavailability [82]. In addition, aldosterone increases ROS production [16], which attenuates insulin signaling by impairing NOS activity and reducing NO bioavailability. Accordingly, superoxide anion (^-^O_2_) interacts with NO, forming peroxynitrite (-ONOO), which has less ability than NO to induce relaxation. Also, the overproduction of ROS leads to oxidation of tetrahydrobiopterin (BH_4_), which is an essential cofactor for eNOS. The reduction of BH_4_ converts eNOS to a ^-^O_2_- producing enzyme, leading to reduced NO release and enhanced oxidative stress [83]. This phenomenon impairs insulin signaling and insulin-induced vascular relaxation. Aldosterone also leads to proteosomal degradation of IRS-1, increasing IGF-1 signaling, which attenuates insulin-induced Akt phosphorylation and NOS activation, and decreases glucose uptake in VSMC [30, 70, 71, 84, 85].

Sherajee and colleagues determined the effects of insulin on Akt phosphorylation in aortas from control rats and rats treated with aldosterone plus salt. Incubation with insulin for 30 minutes increased Akt phosphorylation in aortas from the control group, but was significantly less in aortas from rats treated with aldosterone plus salt, indicating that insulin effects are attenuated by aldosterone actions on Akt signaling. Decreased Akt phosphorylation leads to decreased NOS activity and, consequently, to reduced NO bioavailability. Treatment with spironolactone and tempol recovered Akt phosphorylation, reinforcing the involvement of ROS on aldosterone-induced impairment of insulin signaling [70, 71, 86].

Although MR signaling has been discussed as the primary responsible in outcomes related to diabetes and obesity, it is important to recognize that others mediators, including cytokines, also contribute to vascular insulin resistance. Thus, aldosterone and MR binding, synergistically, converge to effects from others pathways [40].

**Figure 1:**
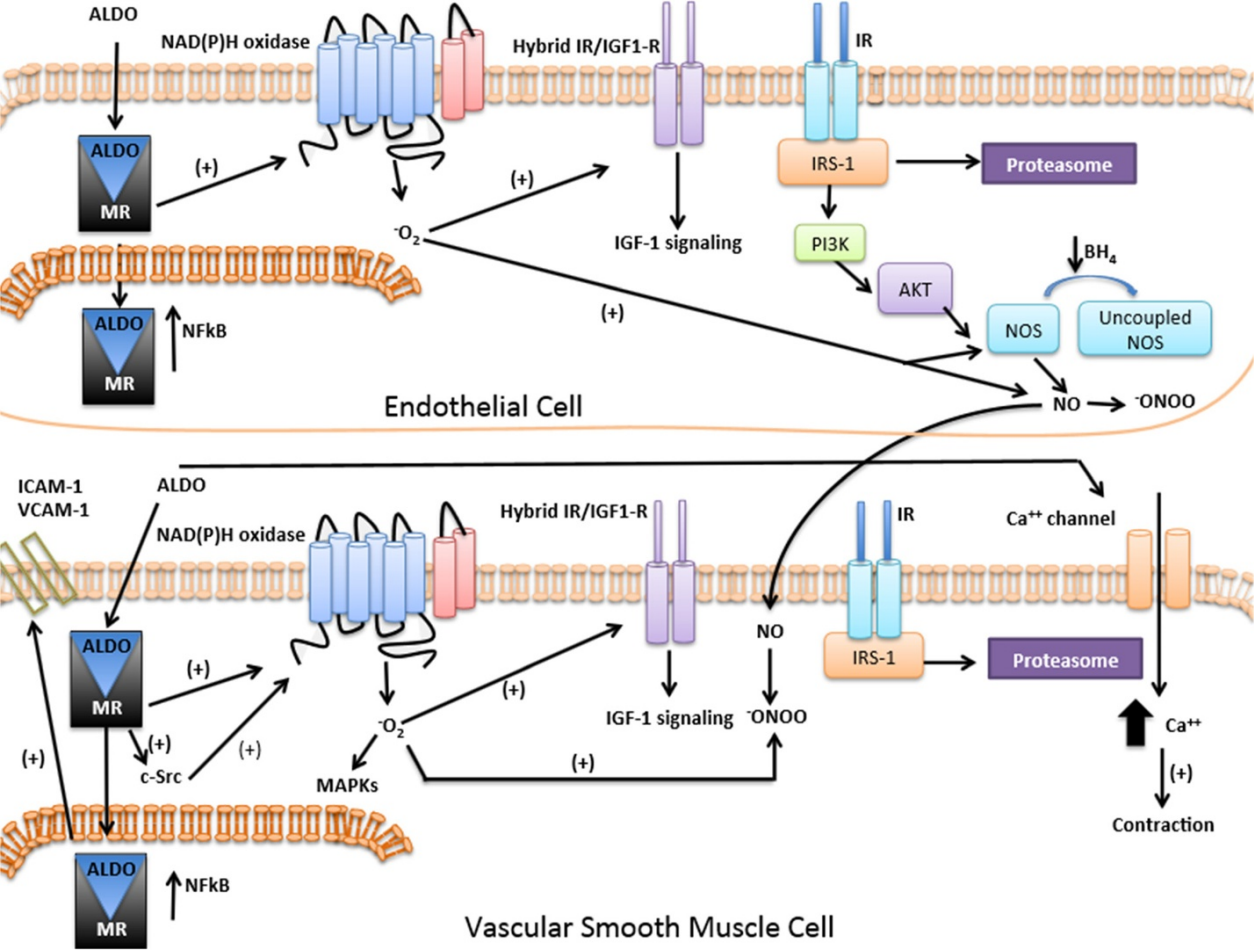
**Effects of aldosterone in vascular signaling.** Aldosterone activates several pathways in the vasculature, both in endothelial and vascular smooth muscle cells, that interfere with insulin signaling. Aldosterone activates NAD(P)H oxidase-dependent reactive oxygen species (ROS) generation or, more specifically, superoxide anion (O_2_^-^) generation, which interacts with nitric oxide (NO) forming peroxinitrite (^-^ONOO). Aldosterone reduces tetrahydrobiopterin (BH_4_), which is an essential NOS cofactor, leading to reduced NO release and impaired vascular relaxation. Aldosterone stimulates mitogen-activated protein kinases (MAPKs) phosphorylation, which leads to activation of proliferative, migratory and inflammatory pathways. Aldosterone activates the formation of hybrid receptors between insulin receptor (IR) and Insulin like Growth Factor-1 receptor (IGF-1R) and induces proteasomal degradation of insulin receptor substrate-1 (IRS-1), decreasing Akt phosphorylation and nitric oxide synthase (NOS) activation. Aldosterone also increases the expression of adhesion molecules, such as vascular cell adhesion molecule 1 (VCAM-1) and Intercellular Adhesion Molecule 1 (ICAM-1), and activates transcription factors, including Nuclear Factor Kappa B (NFkB). Aldosterone increases calcium (Ca^+2^) influx, further decreasing vascular relaxation and favoring contractile responses. (+) indicates activation; (-), inhibition.

In summary, substantial evidence indicates that aldosterone plays a key role in CVD, with MR antagonists representing potential excellent tools to minimize the risks of CVD. Moreover, both obesity and DM2 are associated with elevated plasma aldosterone levels and vascular abnormalities. Vascular insulin resistance is considered an early contributor to vascular damage [40]. Some of the mechanisms by which aldosterone interferes with insulin signaling and induces vascular dysfunction in obesity and DM2 are already clarified and include increased IGF-1R expression; augmented hybridization of IGF1-R and IR, which are dependent on the degradation of IRS-1 by the proteasome; decreased NO bioavailability and Akt phosphorylation. Interestingly, ROS generation modulates the activity/expression of most of these potential players in insulin resistance, strengthening the concept that aldosterone-induced oxidative stress impairs vascular insulin signaling Figure 1. However, more studies are necessary to find other contributing mechanisms to aldosterone-induced vascular dysfunction in obesity and DM2.
